# Impact of Chloramination on the Development of Laboratory-Grown Biofilms Fed with Filter-Pretreated Groundwater

**DOI:** 10.1264/jsme2.ME12095

**Published:** 2012-10-31

**Authors:** Fangqiong Ling, Wen-Tso Liu

**Affiliations:** 1 Department of Civil and Environmental Engineering, University of Illinois at Urbana-Champaign, 205 N. Mathews Ave., Urbana, Illinois 61801–2352, USA

**Keywords:** chloramine, drinking water, biofilm, 16S rRNA pyrosequencing

## Abstract

This study evaluated the continuous impact of monochloramine disinfection on laboratory-grown biofilms through the characterization of biofilm architecture and microbial community structure. Biofilm development and disinfection were achieved using CDC (Centers for Disease Control and Prevention) biofilm reactor systems with polyvinyl chloride (PVC) coupons as the substratum and sand filter-pretreated groundwater as the source of microbial seeding and growth nutrient. After 2 weeks of growth, the biofilms were subjected to chloramination for 8 more weeks at concentrations of 7.5±1.4 to 9.1±0.4 mg Cl_2_ L^−1^. Control reactors received no disinfection during the development of biofilms. Confocal laser scanning microscopy and image analysis indicated that chloramination could lead to 81.4–83.5% and 86.3–95.6% reduction in biofilm biomass and thickness, respectively, but could not eliminate biofilm growth. 16S rRNA gene terminal restriction fragment length polymorphism analysis indicated that microbial community structures between chloraminated and non-chloraminated biofilms exhibited different successional trends. 16S rRNA gene pyrosequencing analysis further revealed that chloramination could select members of *Actinobacteria* and *Acidobacteria* as the dominant populations, whereas natural development leads to the selection of members of *Nitrospira* and *Bacteroidetes* as dominant biofilm populations. Overall, chloramination treatment could alter the growth of multi-species biofilms on the PVC surface, shape the biofilm architecture, and select a certain microbial community that can survive or proliferate under chloramination.

Drinking water distribution systems (DWDS) represent a large part of the infrastructure of the drinking water industry. The vast surface of these systems is in contact with bulk liquid and can support the growth of microorganisms, which further leads to the formation of biofilms. Biofilm formation can have negative impacts on drinking water quality during distribution, and is considered an unknown factor in risk assessment of drinking water consumption (41). Post-disinfection is a common practice across the world to prevent biological deterioration of drinking water quality in DWDS; however, biofilms, which consist of multiple species, can still be observed on the surface of pipe sections (34) and water meters (14) in DWDS even in the presence of disinfectants. Thus, understanding how microbial biofilms can survive under disinfection treatment is important to improve and possibly predict the performance of DWDS, as well as to better assess the risks associated with drinking water.

Studies have been carried out to understand how biofilms resist treatments. It is hypothesized that biofilm matrix plays an important role in reducing the penetration of disinfectants, to a level that the penetrated disinfectants can no longer inactivate cells deep inside the biofilm matrix (38). This hypothesis is supported by studies using free chlorine and monochloramine, which exhibited different penetration profiles into biofilm matrices (6, 9, 24). It was further observed (21) that cells embedded in biofilms were 10–1,000 times more resistant to free chlorine disinfection than free-living cells. Likewise, biofilm cells under chloramination could lose respiratory activities in a non-uniform manner, with the greatest inactivation or lysis occurring during the direct contact of surface biofilms with disinfectant-containing bulk liquid (15). Biofilms with different microbial compositions could also exhibit different levels of resistance to disinfectants (36); however, possibly due to the diffusional limits of disinfectants and short periods of disinfection treatments (*e.g.*, 2–48 h), no significant changes in the architecture and community structure of biofilms were reported (15, 24).

In DWDS, disinfectants are usually in contact with biofilms for an extended period to achieve complete penetration of monochloramine. This can result in a more uniform profile of disinfectant concentration within biofilms, especially for biofilms growing on non-reacting substrata such as glass (23) and polycarbonate (24). This further suggests that the diffusional limitation of disinfectants may not be the sole reason why biofilms could survive in disinfectant-containing environments, and short-term disinfection experiments are not sufficient for understanding the biofilm response to disinfection. It is reported that community succession and architectural development could take place during biofilm growth in oligotrophic environments (28); however, it is not clear how disinfection will influence these processes. So far, studies that investigated the change of the microbial community under long-term disinfection treatments have been mainly conducted in large settings (20, 30, 32) which, due to the difficulty of achieving good experimental controls and sampling, could not resolve the likely different influence of growth and disinfection. Thus, our main objective was to determine the impact of disinfection on the development of microbial biofilms grown under an oligotrophic environment for up to 10 weeks. This allowed us to observe biofilm growth during successional development, and to examine and compare the development of the biofilm architecture and microbial community with or without the influence of monochloramine disinfection (or chloramination). We used groundwater pretreated with sand filter to mimic the growth of biofilms in oligotrophic environments in water distribution systems. This was based on the consideration that the majority of the small towns in the Midwest region of the U.S.A. use groundwater as source water and only apply simple physical and chemical treatments with residual disinfectants before the treated water is distributed to the network (42).

## Materials and Methods

### Biofilm reactor setup and operation

Experiments were conducted under the same operational conditions twice to observe the development of biofilms in the presence and absence of disinfection treatments. CDC (Centers for Disease Control and Prevention) biofilm reactor systems (Biosurface Technologies, MT, USA) (12) with polyvinyl chloride (PVC) coupons as the substrata were used to grow biofilms. The reactor configurations are shown in Fig. S1. In each experiment, two reactors, which were used in parallel as a control and treatment, were fed with pre-treated groundwater for 2 weeks to allow initial development of biofilms to a level that could provide enough biomass for quantitative imaging analysis and microbial community analysis. After this point, the biofilms were further grown for 8 weeks under natural development (control reactor) or subjected to monochloramine treatment (treatment reactor). The groundwater used was collected from Newmark Civil Engineering Laboratory building at the University of Illinois and pretreated with greensand filter to remove manganese and iron. During the operation, the system was completely covered with aluminum foil to prevent the growth of phototrophic bacteria.

To start chloramination, the water in the CDC reactors was decanted at the end of week 2 and filled with chloraminated groundwater. The reactor was continuously fed with chloraminated groundwater at a constant flow rate of 2.6 mL min^−1^ for 8 more weeks. The chloraminated groundwater was prepared by adding 35 mL monochloramine stock solution into one liter of groundwater to achieve a targeted combined chlorine concentration of 10 mg Cl_2_ L^− 1^. The monochloramine stock solution was freshly prepared by mixing chlorine (sodium hypochlorite; Sigma-Aldrich, St Louis, MO, USA) and ammonia (ammonia chloride; Ricca Chemical Company, Arlington, TX, USA) at a weight ratio of 5:1 for 5 min.

### Water chemistry analysis

Concentrations of total and free chlorine were measured using an N,N-diethyl-*p*-phynylenediamine (DPD) chlorine test kit (Hach, Loveland, CO, USA). Non-volatile organic carbon was measured with a Tekmar Dohrmann Apollo 9000 HS carbon analyzer (Teledyne Takmar, Mason, Ohio, USA) based on Standard Method 5310B (7). Metal analyses were performed using a Varian Vista Pro CCD simultaneous inductively coupled plasma optical emission spectrometer (Varian, Palo Alto, CA, USA) by the Center for Chemistry and Technology at Illinois State Water Survey (http://www.isws.illinois.edu/chem/ias/, Champaign, IL, USA). Nitrate and sulfate were analyzed with Dionex DX-500 and ICS-5000 ion chromatographs (Thermo Fisher Scientific, CA, USA) respectively. Nitrite, ammonia, and orthophosphate analyses were performed using the automated colorimetric method published by the Environmental Protection Agency (http://water.epa.gov/scitech/methods/cwa/). pH and DO were measured with an Orion 4 star portable pH DO meter (Thermo Orion, MA, USA).

### Biofilm biomass retrieval and DNA extraction

Biofilm samples on the coupon surface were retrieved from the control and treatment reactors at weeks 2, 6, and 10 for microbial community analysis by considering the number of coupons available within a CDC reactor and the number of CDC reactors used. The coupons were first transferred to a Petri dish containing 5 mL sterile phosphate-buffered saline (PBS) and were then physically scraped with sterile cotton swabs. The cotton swab and the PBS were then collected and transferred to a 50 mL sterile centrifuge tube. This procedure was repeated three times. The centrifuge tube containing the cotton swabs and PBS solutions was vigorously mixed by vortexing. Biomass in the buffer solution was pelleted by centrifugation at 10,000 × *g* for 10 min. The aforementioned step was repeated until turbidity was not observed in the tubes to ensure that the cells trapped in the cotton swabs were eluted into the PBS and precipitated. Prior to the start of disinfection in the third week, microbial cells present in the bulk liquid were obtained by filtering all liquid in the reactor (300 mL) through 0.22 μm polycarbonate filters (Millipore, MA, USA). Samples were preserved at −80°C prior to DNA extraction according to a previously published protocol (35). Briefly, biomass was treated with enzymatic digestion, physical disruption and chemical lysis. Total nucleic acid was extracted with chloroform:isoamyl alcohol (24:1), precipitated with isopropanol, and fractioned with a QIAGEN AllPrep DNA/RNA mini kit to obtain DNA.

### T-RFLP and data analysis

Terminal restriction fragment polymorphism (T-RFLP) was conducted according to a previously published protocol (26). Duplicate PCR reactions were conducted for each DNA template with bacterial primer pair 47F (5′-CYTAACACATGCAAGTCG-3′) (5) and 927R (5′-ACCGCTTGTGCGGGCCC-3′) (2). The PCR products were pooled and digested with mungbean enzyme to remove single-stranded DNA, purified with a Promega Wizard DNA purification kit, and then digested with *Msp*I (New England Biolabs, MA, USA). The selection of the restriction enzyme was based on the comparison of T-RFLP patterns obtained from the use of two different restriction enzyme digestions (Fig. S2). The result was analyzed with Genemapper V 4.0, and peak binning was conducted using the T-RFLPstat program (1). A distance matrix based on Bray-Curtis distance between samples was generated. The similarities among samples were presented by cluster analysis and non-metric multidimensional scaling analysis using PRIMER 6 software (Plymouth Marine Laboratory, UK).

### 16S rRNA gene pyrosequencing and data analysis

Pyrosequencing reactions were carried out with an LIB-L kit (Roche Applied Science, Indianapolis, IN, USA) with primer set 515F-909R targeting 16S rRNA gene sequences (44). PCR products were purified with the Promega Wizard SV gel and PCR clean-up kit (Promega, Madison, WI, USA). The purified products were quantified with a Qubit fluorometer (Invitrogen, Carlsbad, CA, USA). Amplified products were sequenced at W. M. Keck Center for Biotechnology at the University of Illinois using a 454 FLX Genome Sequencer (Roche Applied Science). Results of 16S rRNA gene pyrosequencing were analyzed with a QIIME pipeline (4). Briefly, noise was removed from flowgrams and the uclust algorithm was used for operational taxonomic unit (OTU) picking. A representative set of sequences that contained one sequence per unique OTU was selected. These sequences were aligned against Greengenes imputed core reference alignment using PyNAST with default settings, and chimera sequences were identified with a Chimera slayer and removed. The aligned sequences were further filtered to remove gaps and non-useful information, and used to generate a phylogenetic tree for further analysis. Taxonomy assignment was conducted with an RDP classifier trained by a dataset from Greengenes OTUs at a minimum confidence level of 0.8. After taxonomy assignments, weighted and non-weighted unifrac distances were calculated and used for principal component analysis (PCoA) analysis. Relative abundance of OTUs in each sample was calculated and used to generate a heat map using the Matlab bioinformatics toolbox. The OTU table was also used for ordination analysis using CANOCO (25). The community data were first analyzed with detrended correspondence analysis (DCA) to determine a suitable model and, according to the manual, the longest length of gradient for this set of data (3.742) fell into the range (3–4) in which both linear and unimodal models could be used. Subsequently, canonical correspondence analysis (CCA) was used for analysis for correlation between community and environmental conditions.

### Confocal laser scanning microscopy and image analysis

Bacterial cells in the biofilm were stained with a LIVE/DEAD BacLight Bacterial Viability kit (Invitrogen), containing fluorescence dyes SYTO 9 and propidium iodide (PI), according to the manufacturer’s instructions. Fresh biofilm samples were stained by adding 20 μL working solution to the biofilm and incubating in the dark for 30 min. Stained biofilm samples were visualized with an LSM 710 confocal laser scanning microscope (CLSM) (Carl Zeiss, Oberkochen, Germany). The SYTO 9 and PI signals were scanned using 488-nm and 581-nm lasers, respectively. Four vertical scans were performed to increase the signal-to-noise ratio. Seven images were obtained for each sample at each time point. Extended focus images and vertical cross sections through the biofilm were generated using the IMARIS software package (Bitplane, Zurich, Switzerland). Quantitative analysis of the images was performed with the COMSTAT software package (13), where biomass signals were defined as all connected regions above a defined volume. Biomass volume (biovolume, μm^3^ per μm^2^ area) and average thickness (μm) in the COMSTAT program were calculated based on the SYTO 9 signals. Comparison of biofilm architecture characteristics between any two given samples was conducted with Student’s t-test or the Mann-Whitney test when normality assumption was not met using Origin 8.1 (OriginLab, Northampton, MA, USA).

## Results

### Groundwater quality and operation of CDC reactors

Groundwater was collected at three different times and analyzed (Table 1). Total organic carbon (TOC) as non-volatile organic carbon was the sole carbon source for microbial growth and had an average value of 4.03 mg L^−1^, which was comparable to drinking water TOC levels, ranging from 0.05 mg L^−1^ to 12.2 mg L^−1^ in the United States (22). The groundwater also contained 0.91 mg NH_3_-N L^−1^ and was low in nitrite (0.054 mg NO_2_^−^-N L^−1^), nitrate (<0.07 mg NO_3_^−^-N L^−1^), and orthophosphate (0.029 mg-P L^−1^). Free and total chlorine were not detected in the groundwater.

**Fig. 1:**
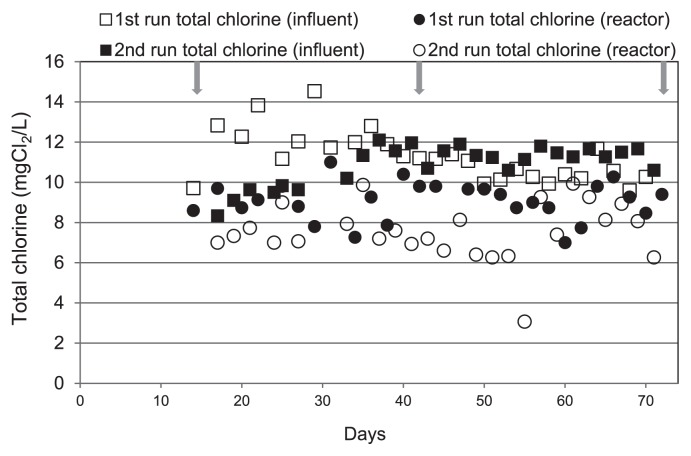
Chlorine levels in treatment reactors during two chloramination experiments. Total and free chlorine levels are presented. Arrows indicate days when biofilm samples were collected. Samples were taken from the control reactors at the same time points.

During operation, the treatment reactor was subjected to 8 weeks of chloramination from the beginning of week 3. As shown in Fig. 1, the total chlorine level in the reactor fluctuated around 9.1±0.4 mg Cl_2_ L^−1^ [95% confidence interval (CI)] and 7.5±1.4 mg Cl_2_ L^−1^ (95% CI) in the first and second runs, respectively. These levels were lower than in the influent, which were 11.3±0.5 mg Cl_2_ L^−1^ and 10.9±0.4 mg Cl_2_ L^−1^ in the first and second runs, respectively. This difference was due to the chlorine consumed by the bulk liquid and biofilms in the reactor. Trace amounts of free chlorine (<6–6.4% total chlorine) detected in both runs were likely due to a test error caused by high concentrations of monochloramine (manufacturer’s manual). The control reactor was fed with groundwater throughout this period. During the experiment, average pH and dissolved oxygen in both reactors were observed to fluctuate around 8.26±0.03 (95% CI) and 7.99±0.09 mg O_2_ L^−1^ (95% CI), respectively.

### Development of biofilm architecture

**Fig. 2:**
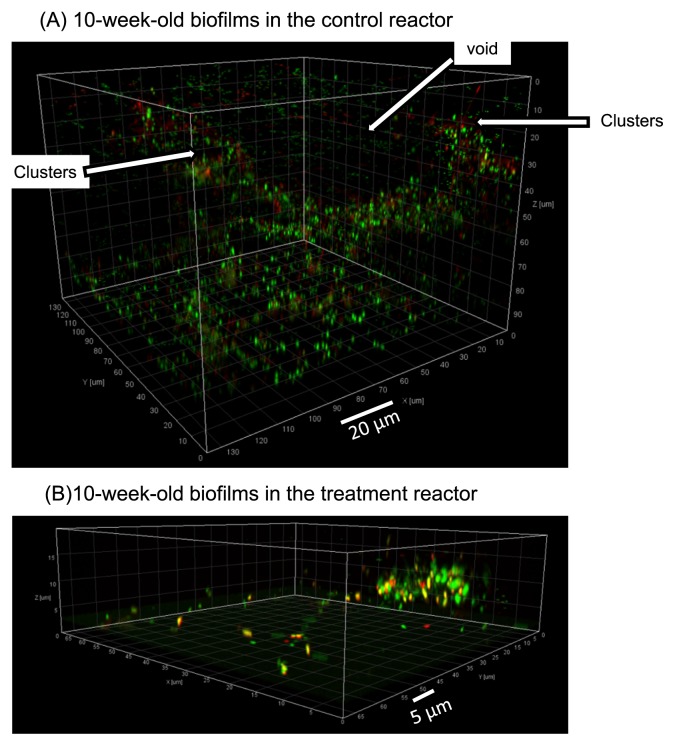
CLSM images showing architecture of 10-week-old biofilms in the control (A) and treatment (B) reactors. Images are presented as 3-D images. Green signals are SYTO-9-stained cells, representing total cells, while red signals are PI-stained cells, representing membrane-damaged cells.

Biofilm samples from the treatment reactors were taken right before, and 4 and 8 weeks after chloramination. The control reactors were sampled at the same time points as the chloraminated reactors. The 6-week-old biofilms from the treatment reactor in the first experimental run could not give a sufficient fluorescence signal after disinfection, using the same level of fluorescence power with the CLSM for the control treatment. By increasing the level of fluorescence, this issue was resolved both in the 10-week-old treated biofilm and in the second experimental run. Fig. 2 shows the IMARIS-reconstructed architecture of biofilms taken from chloraminated and control reactors. The cells in green are membrane-intact cells in contrast to membrane-damaged cells which are stained in red. In the control reactors, biofilms after 10 weeks developed into a thick and complex structure with identifiable cell clusters and pore space (Fig. 2A), whereas in the treatment reactor, a thin and flat biofilm structure was observed (Fig. 2B). In 10-week-old biofilms, the cells appeared to be scattered throughout the biofilms in the control reactor, whereas closely-packed cell clusters were observed in the samples treated with monochloramine.

**Fig. 3:**
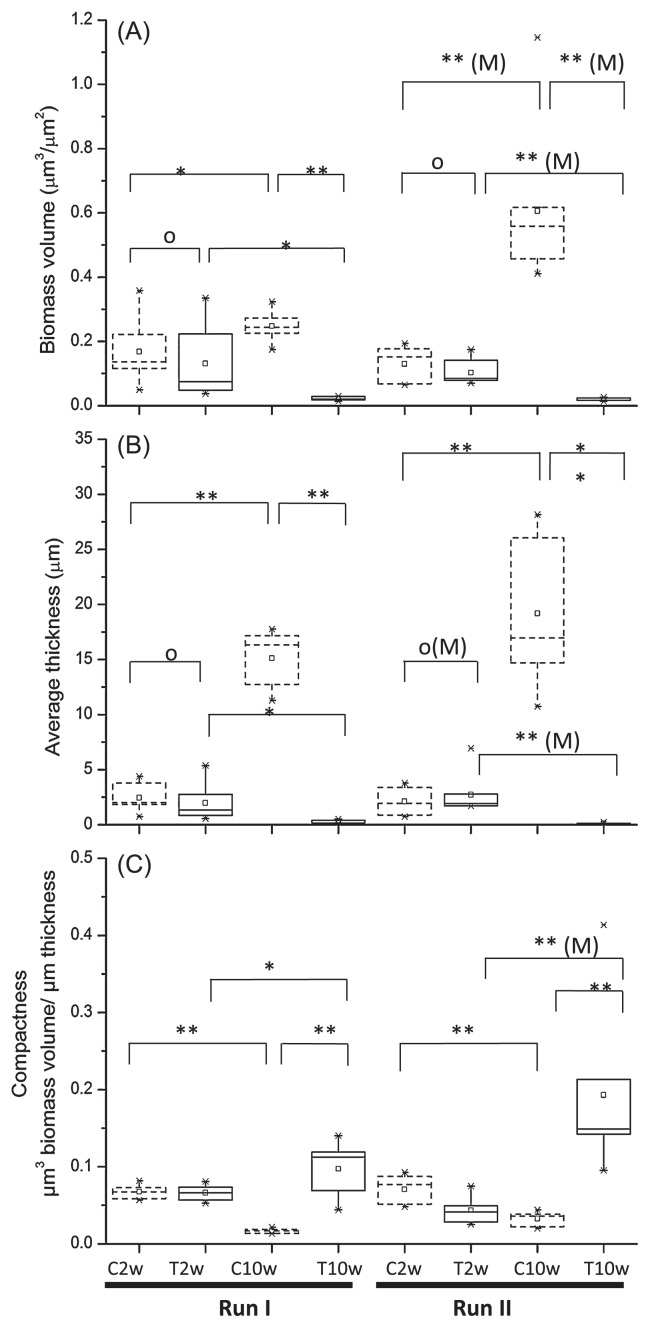
Biofilm architectural characteristics with distributions of biomass volume (A), average thickness (B), and compactness (C) from COMSTAT analysis. Results for 2-week-old (C2 and T2) and 10-week-old (C10 and T10) biofilms from control (C2 and C10) and treatment (T2 and T10) reactors in the first and second run of experiments (denoted as I and II) are presented as boxplots. Lines in box plots present the 25, 50, and 75 percentiles of the distribution and asterisks indicate outliers (n=7). Statistical tests for the difference between two population means were conducted for pairs of samples, indicated by 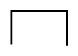. Pairs were tested for whether one of the means was higher than the other. Powers of Student’s t-test or the Mann-Whitney (superscript M) test are shown on top of boxes, with * indicating that *P* falls between 0.01–0.05, and ** indicating *P*<0.01.

The biofilm architecture in the control and chloraminated reactors was further analyzed quantitatively using COMSTAT. Based on the biomass volume and biofilm average thickness obtained, the development of the biofilm architecture exhibited different trends between the treatment and control reactors (Fig. 3). In control reactors, biomass volume increased from 0.17±0.1 to 0.28±0.05 μm^3^ μm^−2^ in the first run, and from 0.13±0.05 to 0.61±0.25 μm^3^ μm^−2^ in the second run; however, in treatment reactors, biomass volume dropped from 0.13±0.11 to 0.02±0.01 μm^3^ μm^−2^ and from 0.10±0.04 to 0.02±0.01 μm^3^ μm^−2^ in the first and second runs, respectively. Biofilm average thickness during biofilm growth was observed to increase and decrease without and with monochloromination, respectively. In the control reactors, biofilm average thickness increased from 2.44±1.24 to 15.13±2.49 μm and from 2.10±1.29 to 19.17±6.36 μm in run 1 and 2, respectively. In the treatment reactors, biofilm average thickness dropped from 1.96±1.67 to 0.27±0.16 μm, and from 2.72±1.90 to 0.12±0.06 μm in run 1 and 2, respectively.

Based on Student’s t-test or the Mann-Whitney test (Fig. 3A and B), the biofilms taken from the control and treatment reactors were not different (*P*>0.4) in biomass volume and average thickness at week 2, but were significantly different (*P*<0.05) at week 10. The biomass volume and average thickness in the control reactor showed a significant increase at week 10 compared to week 2. In contrast, the biovolume and thickness in the treatment reactor at week 10 were significantly lower than at week 2 (*P*<0.05 for each comparison). These results suggest that chloramination could lead to the development of a thinner biofilm with less biomass, and natural development could result in a thicker biofilm with more biomass. It is likely that the thin biofilms observed were a result of biofilm sloughing due to chloramination; however, whether these biofilms could still allow cells in bulk liquid to attach remains to be further studied. The ratio of green and red signals, which were calculated and summarized in Table S1, did not show any dependence on the presence or absence of disinfectants.

### Biofilm community development revealed by T-RFLP

**Fig. 4:**
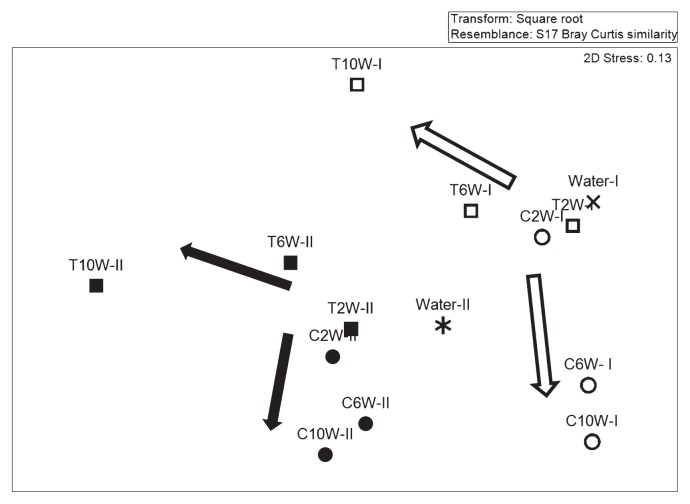
Non-metric multidimensional scaling results from the T-RFLP analysis experiment. Similarity between samples was calculated with the Bray-Curtis distance. C: control reactor; T: treatment reactor. Empty and solid symbols represent biofilm samples from the first and second experimental runs, respectively, and “×” and “*” indicate bulk liquid samples from first and second runs, respectively.

To understand the role of chloramination in microbial community development, T-RFLP was used to track temporal changes of microbial community in the biofilms from two experimental runs. Bulk liquid was sampled immediately before disinfection. T-RFLP fingerprinting patterns showed differences in communities in terms of the shifts in the dominant terminal restriction fragments (T-RFs) and their abundance. Within each run, control and treatment reactors shared common major T-RFs before disinfection started, while the major T-RFs shifted after 8 weeks of disinfection (Fig. S3). Non-metric multi-dimensional scaling analysis was used to plot the biofilm samples on a “map”, where the distance showed the relative similarity of the community structure (Fig. 4). Within each run, the 2-week-old samples from the control and treatment reactors were closely grouped together. Biofilm samples obtained after disinfection were distinctly different from samples of the same age without disinfection, suggesting that the processes of community development under these two conditions were different. Between the two runs, differences in microbial composition were observed in the groundwater fed into the CDC reactors. As a result, differences were observed in the community structures of the microbial biofilms established at weeks 2, 6 and 10 within the same treatment.

### Microbial community assembly in the presence and absence of chloramination

Based on the T-RFLP results, distinctive communities from both runs were analyzed with 16S rRNA gene pyrosequencing. Each sample was analyzed in duplicate using two different barcoded primer sets. After sequence quality processing (*e.g.*, denoising and chimera sequence removal), PCoA based on weighted unifrac distance revealed that different barcoding did not cause a difference in beta-diversity (Fig. S4). Meanwhile, the topology of the PCoA plot agreed with the directional changes of microbial community observed in T-RFLP analysis (Fig. 4).

**Fig. 5:**
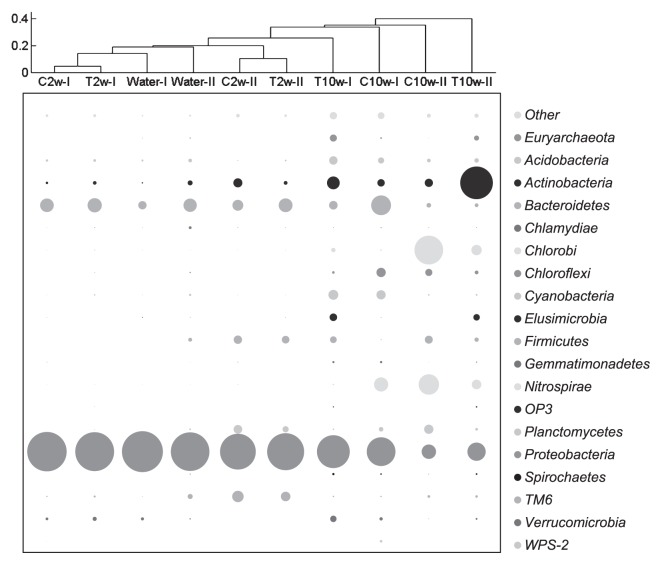
Community compositions of representative biofilm samples from chloraminated and non-chloraminated reactors at phylum level. Relative abundance of each phylogenetic group in a sample was calculated as the mean of the results from replicates.

Community compositions between 2-week-old biofilm samples and 10-week-old biofilms with and without chloramination were further compared by pooling the sequences from the same sample with two different barcodes. At phylum level (Fig. 5), both runs showed that 2-week-old biofilm samples were dominated by *Proteobacteria* (72.4%–88.0%) and *Bacteroidetes* (6.7–11.3%). Ten-week-old biofilm samples showed a decrease in the relative abundance of *Proteobacteria* and an increase in other groups. Groundwater-fed and chloramine-treated reactors showed a different selection of bacterial and archaeal phyla. In groundwater-fed reactors, a common trend was the increase in *Nitrospirae*, which was detected at a low level (<1%) in 2-week-old biofilms and increased in 10-week-old biofilms to 11.2% and 23.5% in first and second runs, respectively. Other than *Nitrospirae*, *Bacteroidetes* increased in first and *Chlorobi* in second runs. For chloramine-treated samples, *Actinobacteria* increased from <5% to 9.1% and 60.3% in first and second runs, respectively. Chloramine-treated samples also showed an increase in *Euryarchaeota*.

Other bacterial phyla appeared higher than 0.1% in at least one sample in the experiment, including *Acidobacteria*, *Chlamydiae*, *Chloroflexi*, *Cyanobacteria*, *Elusimicrobia*, *Firmicutes*, *Gemmatimondetes*, *Planctomycetes*, *Verrucomicrobia*, and candidate phyla WPS-2, TM6 and OP3. In the domain *Archaea*, detectable levels of *Euryarchaeota* and *Crenarchaeota* were observed.

Community compositions of chloraminated and non-chloraminated biofilms were further compared based on the presence/absence or the relative abundance of OTUs (Fig. S5). The 2-week-old communities contained diverse but evenly distributed microbial populations, whereas 10-week-old biofilm communities were dominant by a few groups. Two-week-old biofilm samples from both control and treatment reactors contained a high abundance of *Phenylobacterium* (20.9–26.8% of total sequences), and treated biofilm samples contained *Mycobacterium*-related populations (7.6 and 59.3%) and *Nitrospira*-related microorganisms (5.6% and 22.3%) in the treatment and control reactors, respectively. *Limnohabitans* appeared in all biofilm samples but were in higher abundance in the first experimental run. *Nitrosomonas* was also detected in 2-week-old biofilm samples and 10-week-old biofilm samples, but at low abundance (<1%).

**Fig. 6:**
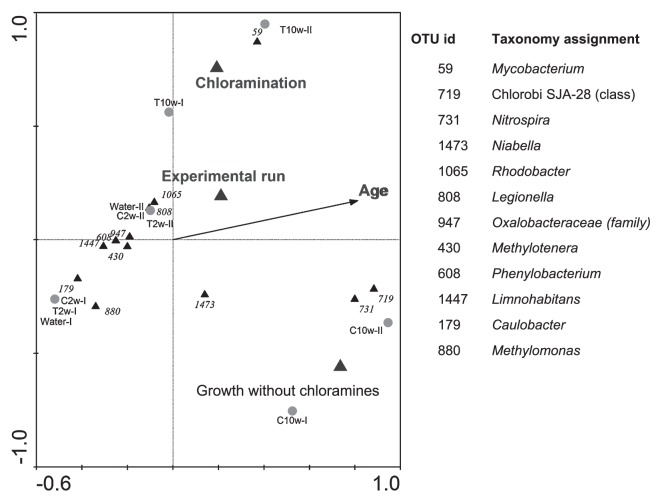
CCA triplot for the correlation between communities and tested factors. OTUs with highest weights (>10%) are plotted on the graph.

Ordination analysis was conducted on the OTUs observed in the pyrosequencing analysis (Fig. 6). Explanatory variables for constrained ordination included chloramination, growth without chloramination, biofilm age, and experimental run. The Monte-Carlo test performed under 499 permutations gives a *P*-value of 0.048 for first canonical axes and 0.002 for all canonical axes. Results are plotted with OTUs weighing >10%, which gave twelve OTUs on the graph. Among these, *Mycobacterium* is strongly correlated with chloramination, and OTUs classified into genus *Nitrospira*, genus *Niabella* (under the order of *Sphingobaceteriales*), and phylum *Chlorobi* were correlated with non-chlorinated and aged biofilm. A few OTUs are plotted close to the origin of the axes, which implies that their appearance could not be explained by the environmental variables tested.

## Discussion

This study examined the effect of chloramination on the development of oligotrophic biofilms over a period of 8 weeks. Our findings revealed that under 8-week disinfection with high concentrations of monochloramine, groundwater biofilm underwent a significant reduction in biomass and thickness (Fig. 3), but still contained membrane-intact cells within biofilms (Fig. 2B). In contrast, biofilm growth without monochloramine treatment was observed to increase in biomass and thickness (Fig. 3). This supports previous observations that biofilms could develop in DWDS supplied with monochloramine (14, 45), and suggests that long-term disinfection with monochloramine could not completely eliminate the growth of microbial cells within the biofilms. The IMARIS reconstructed biofilm images clearly showed a thin and compact architecture after 8 weeks of disinfection, whereas biofilms developed in the same period without monochloramine were observed to contain channels and voids (Fig. 2). These observations were different from the results obtained under short-term monochloramine treatments (*i.e.*, ≤ 24 h) (15, 24), which reported that cells on the periphery of biofilms were inactivated first, and no significant sloughing was observed. Based on this, we speculate that when biofilms were subjected to continuous disinfection treatments, cells at the periphery of the biofilm could lose their activities first, and then detach. As a result, the biofilms eventually became thinner and more compact together with a shift in the microbial community (see below). As with biofilm volume and biofilm thickness, biofilm compactness, which we define as the ratio of biomass volume and thickness, could be used to compare the differences of biofilm architecture developed in the presence and absence of disinfection (Fig. 3C).

With respect to the microbial community structure, we observed the succession of microbial communities in control reactors without chloramination. At week 2, a high abundance of *Phenylobacterium* and *Limnohabitans* was observed, which are reported to grow chemoorganotrophically (11, 16, 17). In 10-week-old biofilms, however, the dominant population was shifted to *Nitrospirae*, which are known to consist mostly of uncultured nitrite-oxidizing bacteria that grow chemolithoautotrophically on nitrite and CO_2_. (27) *Nitrospira* have also been reported to benefit from simple organic compounds, yet it is not clear if they were used as an energy source or carbon source (27, 29). This population shift could reflect a change in the metabolic profiles of the multispecies biofilms during their development. Moreover, we detected microorganisms capable of utilizing methyl compounds (*i.e.*, *Methylotenera* and *Methylomonas*) throughout all the samples at various abundances. Their growth was likely due to the presence of detectable methane in the groundwater, and agreed with the detection of methylotrphs in water meter biofilms in the local drinking water distribution systems where groundwater was supplied as source water (14). Inter-run differences in the community structures were also observed. For example, an OTU related to phylum *Chlorobi* had high abundance in the 10-week-old biofilms from the second reactor run. Members of this phylum are widely spread in anoxic and anaerobic environments based on different metabolic growth (phototrophic, non-phototrophic, heterotrophic and autotrophic) (3). Thus, it is unclear why they became dominant in aged groundwater biofilms.

Our findings further revealed that prolonged chloramination could act as a selective environmental factor or an allogenic factor that altered the microbial community composition in biofilms during microbial succession. The abundance of several microbial populations was shown to be chloramination dependent. For example, *Nitrospirae*-related populations were primarily observed in unchloraminated biofilms in this study and in pretreatment processes of drinking water production and in non-disinfected DWDS (10, 28, 33), suggesting that they were sensitive to chloramination or disinfection treatments. In contrast, an increase in the abundance of phylum *Actinobacteria* or *Mycobacterium*-related populations was shown by canonical analysis to be strongly correlated with chloramination. This observation agreed closely with previous findings on the detection of *Mycobacterium-*related microorganisms in disinfected DWDS using cultivation and molecular-based detection methods (18, 43). Another OTU, classified into genus *Limnohabitans* in family *Comamonadaceae* was also observed to occupy 14.9% of the 10-week-old chloraminated sample in the first experimental run, and was present in the second experimental run. The family *Comamonadaceae* was observed in a chlorinated PVC pilot system by isolation (31) in a distribution system simulator receiving chloraminated tap water by clone library (45), and in a water meter biofilm receiving disinfected groundwater (14).

The reason why *Mycobacterium*-related species can survive and possibly proliferate under disinfection treatments is likely associated with their ecological and physiological traits of biofilm formation, amoeba-associated lifestyle (40), and resistance to disinfection (39). So far, the cell wall of *Mycobacteria* is known to contain mycolic acids that are hydrophobic and resistant to the penetration of disinfectants (8). *Mycobacterium* cells such as *Mycobacterium avium* and *Mycobateirum intracellulare* can readily form aggregates, and have been shown to be more resistant to free chlorine when they are in a biofilm lifestyle than in a planktonic lifestyle (37). It is suspected that low-nutrient conditions can also enhance disinfectant resistance, and this has been observed in microorganisms from many different taxonomic groups isolated from drinking water or treatment processes, including *Legionella pneumophila*, *Flavobacterium sp.*, *Klebsiella pneumonia* (20) and *Mycobacterium gordonae* (19); however, the reason for the survival of *Comamonadaceae* in disinfected water has not been specifically tested and reported, and should be investigated.

In summary, disinfection conditions with a high disinfectant concentration and a long contact time could control the growth of multi-species biofilms on the PVC surface with a thin and compact structure, but not eliminate them. Chloramination can act as an allogenic factor to shape community development and select a certain community. Some of the populations selected, such as *Mycobacterium*, are of public health importance. These findings are important to better understand biofilm growth in chloraminated drinking water distribution systems.

## Supplementary Material



## Figures and Tables

**Table 1:** Groundwater quality

Parameter	Concentration (mg L^−1^)
Ca (total)	58.8 (±3.6)
Fe (total)	0.62 (±0.36)
Mg (total)	24.8 (±1.5)
Mn (total)	3.33 (±1.48)
orthophosphate-P	0.03 (±0.01)
NVOC	4.03 (±0.77)
NH_3_-N	1.09 (±0.19)
NO_2_^−^-N	0.05 (±0.08)
NO_3_^−^-N	<0.07
