# Supplementary material for: Impact of Chloramination on the Development of Laboratory-Grown Biofilms Fed with Filter-Pretreated Groundwater
Source: Microbes Environ. 2012 Oct 31;28(1):50–7. doi: 10.1264/jsme2.ME12095 (PMC4070696; doi:10.1264/jsme2.ME12095)
Supplement: Supplementary file 1 [file 28_50_s1.pdf]

Figure S1. Reactor configuration.

(A) Reactor and reservoir

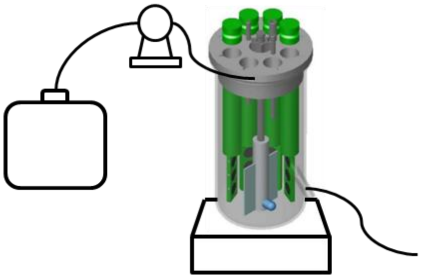

(B) CDC reactor

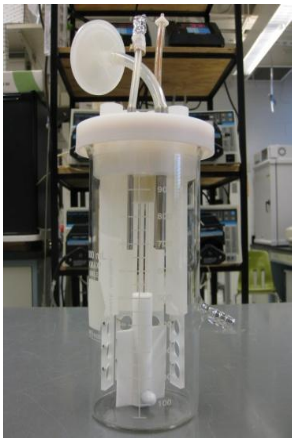

(C) Coupon holder

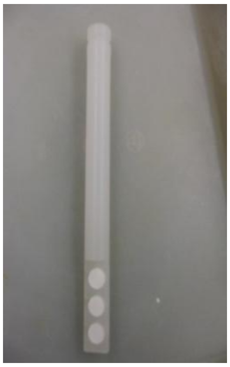

Figure S2. Comparison of MspI and HhaI on digestion of biofilm samples before and after chloramine disinfection.

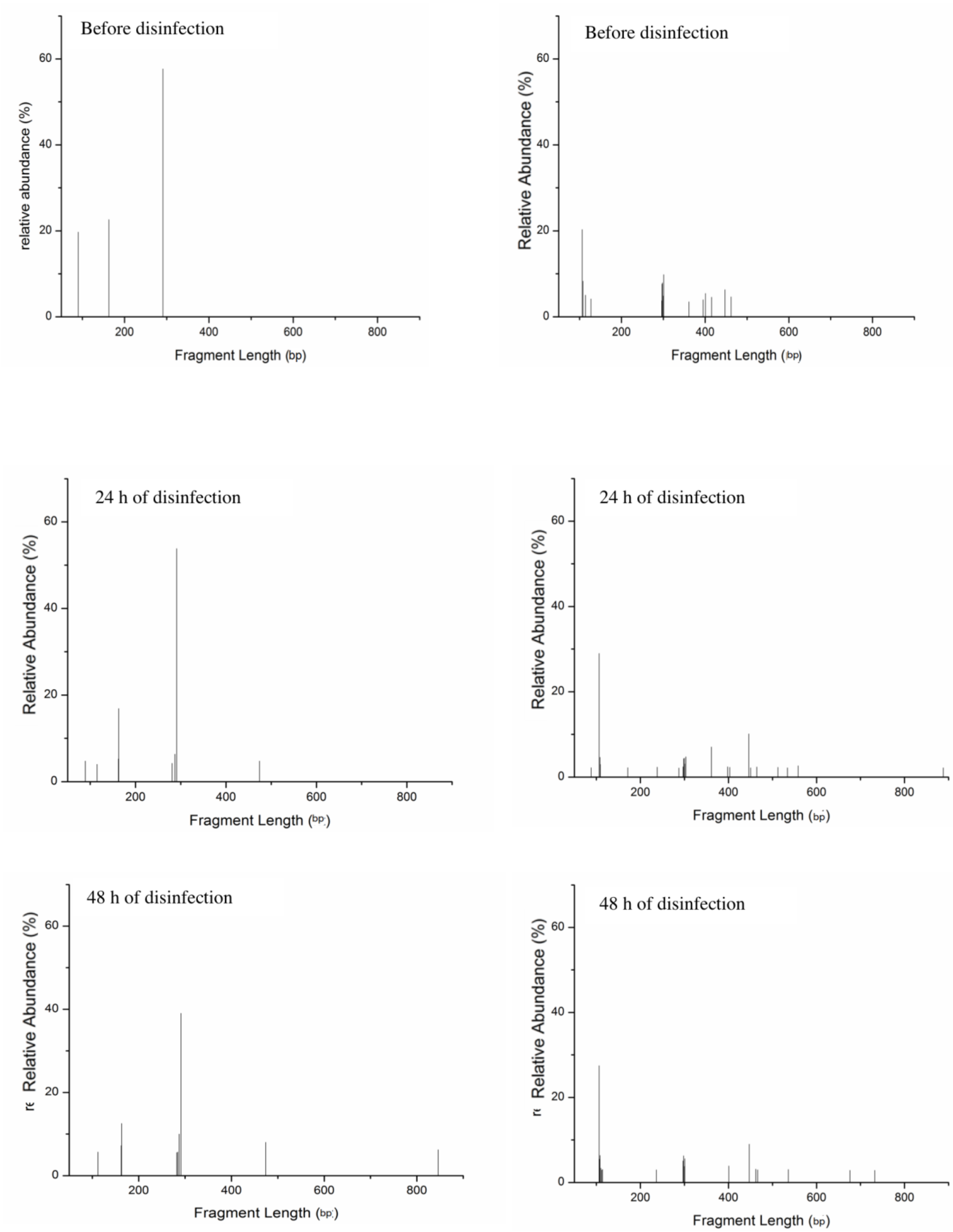

Figure S3. T-RFLP fingerprints from the 10-week experiment. Samples were collected from control (A, C, and E) and treatment reactor (B, D, and F) after 2 weeks (A and B), 6 weeks (C and D), and 10 weeks (E and F) of operation. Peaks marked in the figure are dominant peaks present in one or more samples.

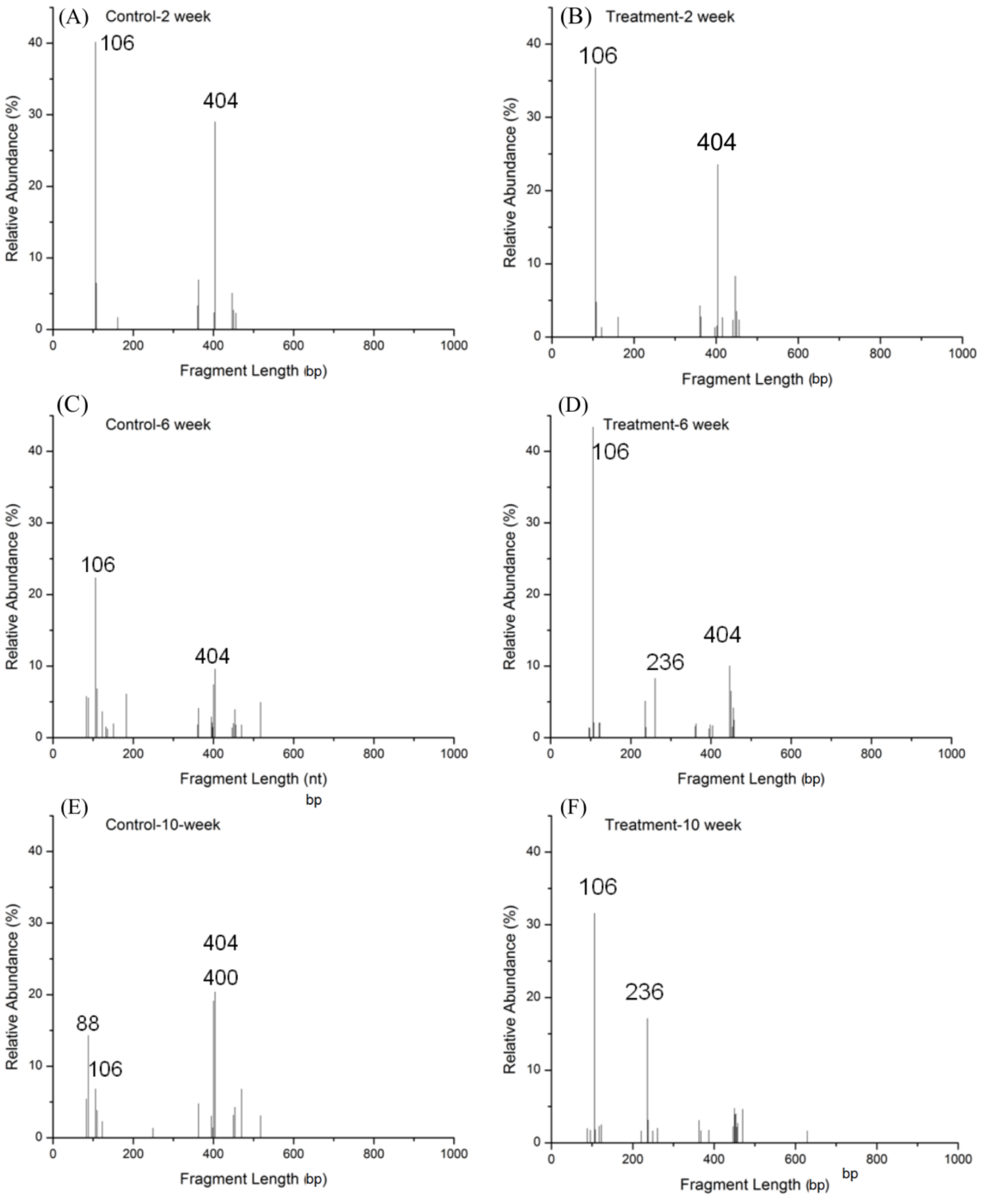

Figure S4. 2-D discrete PCoA analysis based on weighted unifrac distance of biofilm and bulk liquid samples. Dots in the sample color are duplicate barcoded sample from the same one.

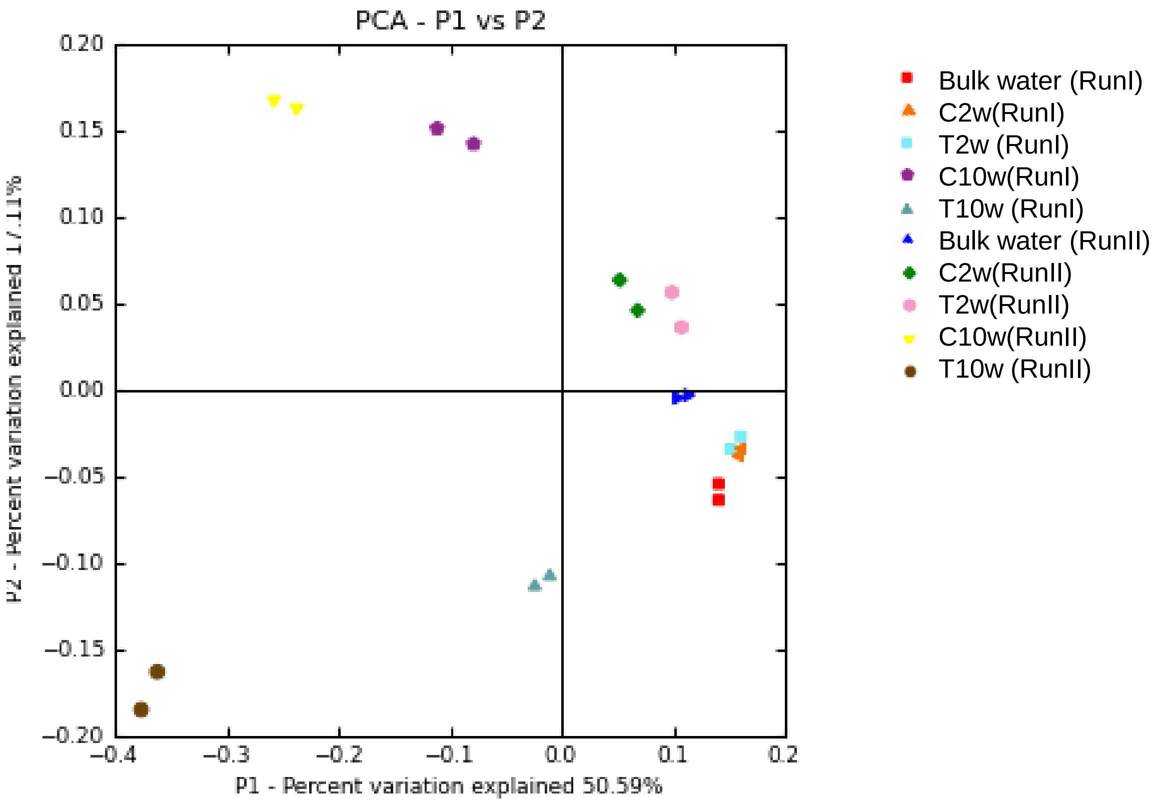

Figure S5. Community compositions of representative biofilm samples from chloraminated and non-chloraminated reactor . OTUs occupying 1% in a certain sample are plotted on the graph. Relative abundances of each phylogenetic group in a sample was calculated as the mean of the results from replicates. OTUs highlighted in yellow are discussed in the text.

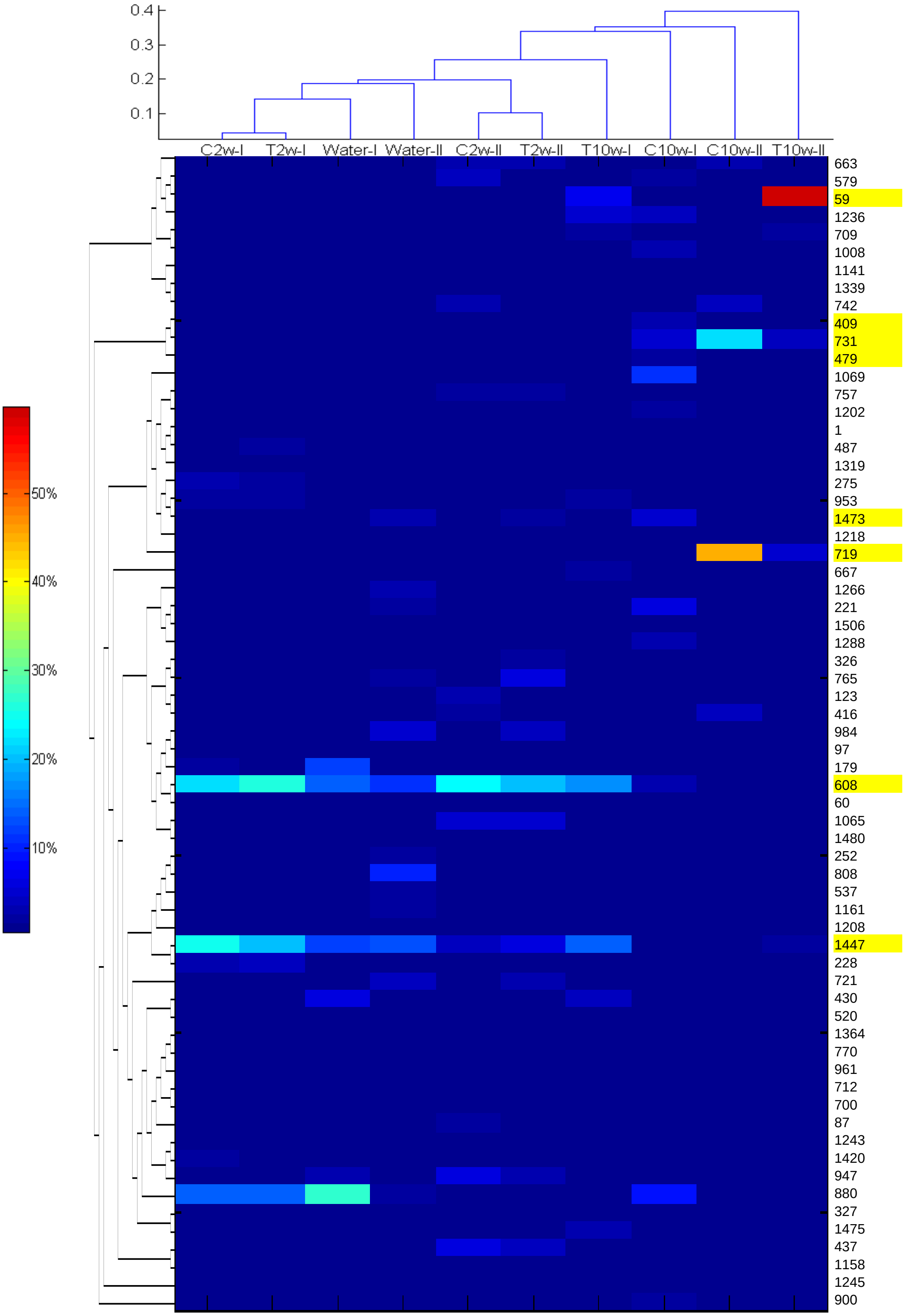

59	*Mycobacterium*	1473	*Niabella*
409	*Nitrospira*	719	*Chlorobi; Class SJA-28*
731	*Nitrospira*	608	*Phenylobacterium*
479	*Nitrospira*	1447	*Limnohabitans*

Table S1. Green/Red ratio in confocal laser scanning microscopy

Experimental Run	Treatment	Ratio of the means of biomass volume between Green and Red signals	
		2-week old	10-week old
I	Control	3.60(1.05)	1.59(0.61)
	Treatment	4.09(1.41)	1.19(0.22)
II	Control	1.52(0.30)	1.40(0.32)
	Treatment	1.21(0.35)	1.11(0.37)
